# Flying over Eurasia: Geographic Variation of Photoperiodic Control of Nymphal Development and Adult Diapause Induction in Native and Invasive Populations of the Brown Marmorated Stink Bug, *Halyomorpha halys* (Hemiptera: Heteroptera: Pentatomidae)

**DOI:** 10.3390/insects13060522

**Published:** 2022-06-04

**Authors:** Dmitry L. Musolin, Margarita Yu. Dolgovskaya, Vilena Ye. Zakharchenko, Natalia N. Karpun, Tim Haye, Aida Kh. Saulich, Sergey Ya. Reznik

**Affiliations:** 1 Department of Forest Protection, Wood Science and Game Management, Saint Petersburg State Forest Technical University, Institutskiy Per. 5, 194021 St. Petersburg, Russia; nkolem@mail.ru; 2 Zoological Institute of the Russian Academy of Sciences, Universitetskaya Nab. 1, 199034 St. Petersburg, Russia; bcongroup@gmail.com (M.Y.D.); reznik1952@mail.ru (S.Y.R.); 3 Federal Research Centre the Subtropical Scientific Centre of the Russian Academy of Sciences, Yana Fabritsiusa Str. 2/28, 354002 Sochi, Russia; vilena.p2016@mail.ru; 4 CABI, Rue des Grillons 1, 2800 Delemont, Switzerland; t.haye@cabi.org; 5 Department of Entomology, Saint Petersburg State University, Universitetskaya Nab. 7–9, 199034 St. Petersburg, Russia; 325mik40@gmail.com

**Keywords:** brown marmorated stink bug, diapause, development, geographic variation, *Halyomorpha halys*, invasive pests, microevolution, photoperiodism, photoperiodic response, reproduction

## Abstract

**Simple Summary:**

The brown marmorated stink bug is an invasive true bug that originates in eastern Asia and is considered now one of the most harmful invasive insect pests in North America and Europe. Similar to the many species that produce more than one generation per year, this bug responds to day length: under short-day conditions (which predict the approaching of autumn), adults form a special overwintering (diapause) physiological state, whereas, under long-day conditions (typical for summer), they reproduce. Critical day length is the condition that induces diapause in 50% of adults. This critical day length is usually strongly correlated with the latitude of the population origin. In this study, we compared the critical day lengths of one native (Andong, South Korea) and three invasive (Torino, Italy; Basel, Switzerland; and Sochi, Russia) populations. The critical day lengths of both sexes fell between 14.5 and 15.0 h in the Korean population, and between 15.0 and 15.5 h in the three European populations. The results demonstrate that microevolution was possibly ‘too slow to keep up’ with the rapid spread of the invader across Eurasia. It is expected that in the near future, the critical day length of invasive *H. halys* populations will gradually change to adapt better to the local conditions.

**Abstract:**

Facultative winter adult diapause in *Halyomorpha halys* is regulated by a long-day photoperiodic response. Day length also influences nymphal development, which slows down at the critical (near-threshold) day lengths. We compared the photoperiodic responses of one native (Andong, South Korea) and three invasive (Torino, Italy; Basel, Switzerland; and Sochi, Russia) populations in a laboratory common-garden experiment. Nymphs developed and emerging adults were reared at 24 °C in a range of photoperiods with day lengths of 14.0, 14.5, 15.0, 15.5, and 16.0 h. The critical day lengths of the photoperiodic responses of both sexes fell between 14.5 and 15.0 h in the native Korean population and between 15.0 and 15.5 h in three invasive European populations. The differences between the three invasive populations were not significant, despite their distant origins. Moreover, the difference between the Korean and European populations was much smaller than was expected. The microevolution was possibly ‘too slow to keep up’ with the rapid spread of the invader across Eurasia. It is expected that soon the critical day length of the invasive *H. halys* populations will gradually change to adapt better to local conditions. At present, the critical day length for diapause induction of 15 h 15 min can be used to model the phenology, further spread, and response to climate change for all European populations of the pest.

## 1. Introduction

Biological invasions cause substantial economic losses and represent serious threats to the natural biodiversity [1,2,3,4,5,6]. On the other hand, these unintentional ‘natural experiments’ [7] offer unique opportunities to study processes of microevolution in real time. Any insect species that disperses out of its native range will face new environmental conditions, and a quick adaptation is a necessary requirement for becoming a successful invader [8,9,10]. Therefore, studies of insect invasions are currently among the most important and demanded directions of research in both fundamental and applied entomology.

Seasonal variation in environmental conditions is an essential component of most natural habitats. One of the most common seasonal adaptations in insects is a facultative diapause induced by environmental cues before the beginning of the adverse season. In particular, photoperiodic control of winter diapause induction and termination is based on the natural correlation between seasonal changes in day length and other environmental factors (e.g., temperature, precipitation, food availability, etc.). However, the pattern of this correlation depends on geographic location. Accordingly, geographically distant populations of widely distributed insect species often differ in their photoperiodic responses and demonstrate clinal variation. Their critical photoperiod (i.e., day length, which induces diapause in 50% of the population) linearly depends on geographic latitude [8,11,12,13,14,15,16]. For many invasive species, it has been demonstrated that their dispersion outside their native range is often accompanied by changes in their photoperiodic response [17,18,19].

Among the most invasive insects is the brown marmorated stink bug, *Halyomorpha halys* (Stål, 1855) (Hemiptera: Heteroptera: Pentatomidae), which originates in eastern Asia (China, Korea, Japan, Myanmar, Vietnam, and Taiwan). It is now considered one of the most harmful invasive insect pests in North America and Europe [20,21,22,23,24] and was also recently recorded in Russia [24,25,26]. Native and invasive ranges remain disconnected in Eurasia with a lower diversity of haplotypes in Europe than in Asia [22]. *Halyomorpha halys* overwinters as an adult and its reproductive diapause is induced by short photoperiods. Before the invasion of *H. halys* into other parts of the world, the diapause induction was only studied in Japan: in the populations from Toyama Prefecture (likely Kurobe City; 36°52′ N, 137°27′ E) and Nagano Prefecture (likely Obuse-machi town; 36°42′ N, 138°19′ E), the critical photoperiod for ovarian development was between 13.5 and 14.0 h and 14.75 and 15.0 h, respectively [27,28]. Niva and Takeda [29] showed that short photoperiods not only slowed down maturation and induced diapause, but also accelerated nymphal development. More recently, thermal and photoperiodic impacts on the development and maturation of *H. halys* from the invaded area of Sochi (Krasnodar Region of Russia) were investigated [26]. In highly polyphagous species, such as *H. halys* [23], the rate of development and diapause induction often depends not only on the photoperiod and temperature but also on the food type and quality [8,14] which are much more difficult to control and standardize in an experimental set-up. Consequently, the comparison of the correlation between critical day length and latitude obtained from different studies is unreliable, and ideally, populations from several different geographical locations should be studied in one location under the exact same conditions.

Numerous studies have been devoted to modeling seasonal development, voltinism and the reproduction of *H. halys*, however, in most cases, the photoperiodic response was not accounted for or its parameters were considered as constant, i.e., not influenced by temperature or geographic origin of the population [30,31,32,33,34,35,36]. Moreover, geographic variability of the photoperiodic response was not included in the distribution models developed to predict the potential geographic range of *H. halys* and its response to climate change [37,38,39,40,41,42]. However, the available data on *H. halys*, as well as numerous studies of other insect species, suggest that the parameters of the photoperiodic response of the brown marmorated stink bug may vary both in space (the difference between geographic populations) and time (the gradual adaptation of recently established invasive populations to the local environment) [17,18,43].

In the present study, we compared the patterns of photoperiodic effects on the duration of nymphal development and adult diapause induction in four geographically distant populations of *H. halys* from its native and invasive ranges. The fundamental aim of this work was to experimentally test if the difference between photoperiodic responses of individuals from invasive *H. halys* populations would follow the same correlation of geographic latitude as relatively stable native populations of many other widely distributed non-invasive insect species. In addition, the present study aims to obtain more precise data on the critical day length that induces diapause in *H. halys* adults, which could be used to enhance the power of future bioclimatic envelope models to predict the potential distribution and seasonal dynamics of this invasive pest.

## 2. Materials and Methods

### 2.1. Insects

The present study was conducted with laboratory populations originating from *H. halys* nymphs and adults collected in the following four locations:

(1) Andong, South Korea (ca. 36°41′ N, 128°44′ E; 140 m a.s.l.)—30 individuals were collected in July 2019;
(2) Sochi, Krasnodar Krai, Russia (ca. 43°36′ N, 39°35′ E; 50 m a.s.l.)—more than 100 individuals were collected in July through to August 2019;
(3) Basel, Switzerland (ca. 47°33′ N, 07°36′ E; 260 m a.s.l.)—more than 60 individuals were collected in July through to August 2019;
(4) Torino, Italy (ca. 45°02′ N, 07°35′ E; 240 m a.s.l.)—50 individuals were collected in August 2019.

It should be noted, however, that South Korea is a part of the native geographic range of *H. halys*, whereas the other populations represent the invasive range [20,21,22,23,25].

**Figure 1:**
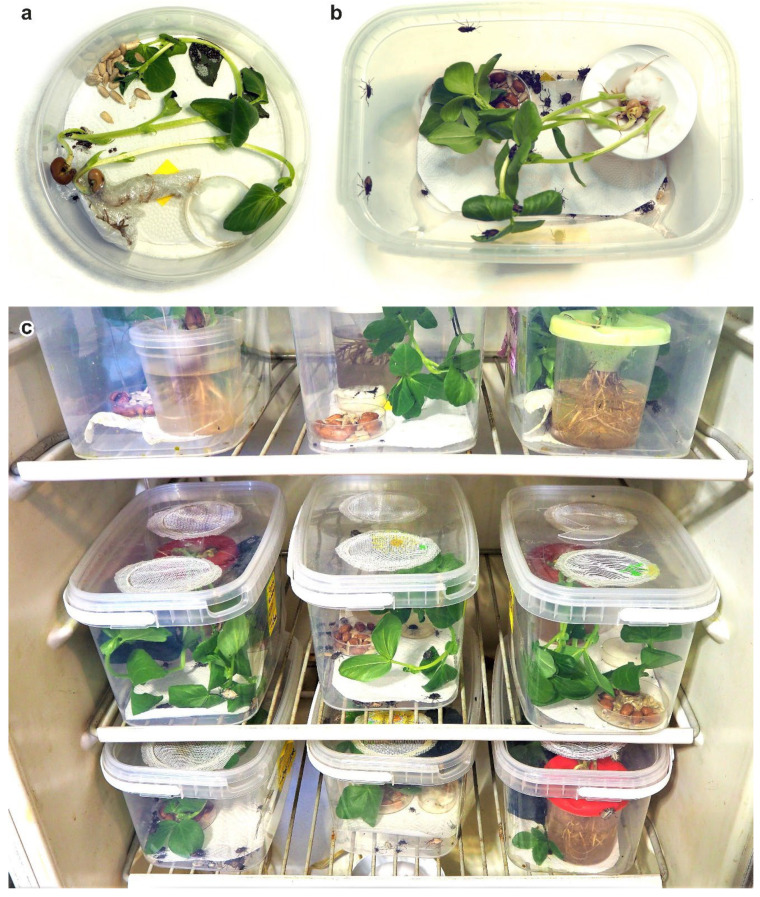
Experimental set-up: (**a**) ventilated transparent plastic containers for rearing *Halyomorpha halys* nymphs and adults during the experiment; (**b**) plastic containers used to support the laboratory culture; (**c**) rearing incubator with pre-set temperature and photoperiod. Photos: K. Samartsev.

Before the experiment was started, bug populations from all four locations were reared for 2 to 3 generations in ventilated transparent plastic containers (28 cm × 19 cm × 14 cm; Figure 1a,b) under laboratory conditions (temperature 25–28 °C, photoperiod L:D 16:8 h). Bugs were fed with peanuts, sunflower seeds, carrots and broad bean seedlings; water was provided in plastic cylinders plugged with cotton balls. During the experiments, the nymphs and adults were reared in ventilated transparent plastic cylinders (12 cm diameter and height) and fed with the same diet as described above (Figure 1).

### 2.2. Nymphal Development

Egg masses no older than 24 h were collected and kept at 24 °C and L:D 16:8 h. The moulting of nymphs from the 1st to the 2nd instar was recorded daily, 4–6 h after switching on the light. Groups of 25 nymphs that had moulted to the 2nd instar within 24 h were randomly chosen and distributed over five experimental treatments with the following photoperiods (L:D): 14:10, 14.5:9.5, 15:9, 15.5:8.5, and 16:8 h. The temperature was kept constant at 24 °C for all treatments. The light regimes were selected based on the results of earlier studies [26,27,28,44]. Fresh food and water were provided, and emerged adults were recorded 3 times a week (i.e., every 2 or 3 days). In total, the duration of development from the 2nd nymphal instar to the moulting to the adult stage was recorded for 1617 adults (55–96 adults from each population per photoperiod). The data for males and females were pooled, as there is no difference in the length of the pre-adult development between sexes [26].

### 2.3. Development of Reproductive Organs and Fat Body

**Figure 2:**
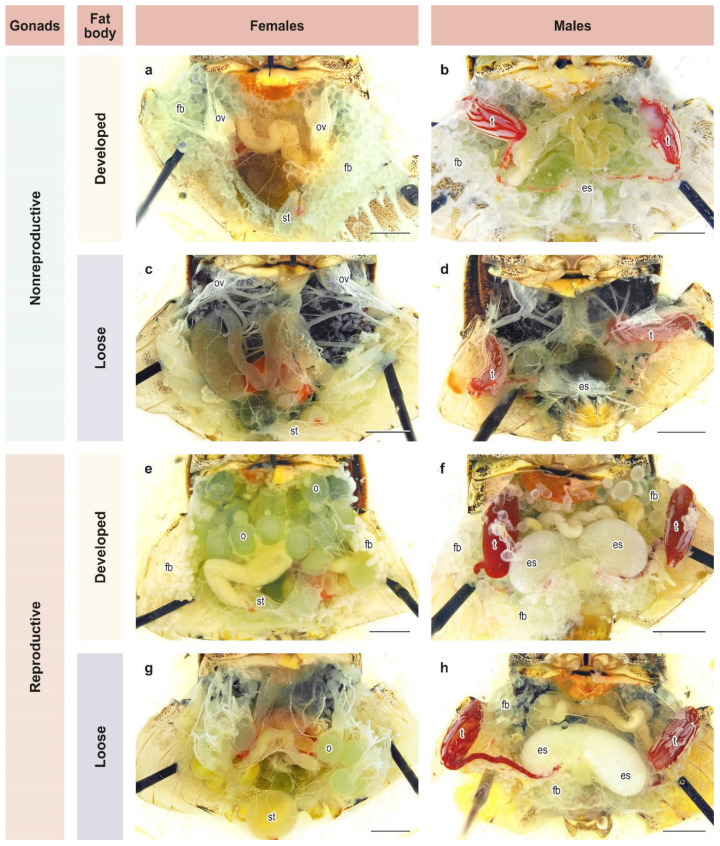
State of development of gonads and fat bodies in females and males of *Halyomorpha halys*. (**a**–**h**) different combinations of states of gonads and fat body in females and males, see text for details. Letters of references: es, ectodermal sacs; fb, fat body; o, ovaries; ov, ovarioles; st, spermatheca; t, testes. Scale bar = 2 mm. Photos: K. Samartsev.

Groups of 3–5 adults of both sexes that had emerged within 2–3 days were placed separately in cylinders and reared further under the same photo-thermal conditions and on the same diet as in the previous experiment. Twenty-five days after emergence, all adults were dissected. This age was chosen based on an earlier study [26] and constitutes approximately 1.5 times the mean period from female emergence to deposition of the 1st egg mass under long-day conditions [45]. At dissection, the reproductive state and fat body development of males and females were evaluated based on the binary scale commonly used for *H. halys* and other pentatomids [26,33,45,46,47,48,49,50,51,52,53]. Adults were considered to be in a reproductive diapause if their gonads were in the nonreproductive state (Figure 2a–d). Otherwise, females with mature eggs or vitellogenic oocytes in their ovarioles (Figure 2e,g) and males with secretory fluids in their ectodermal sacs of the accessory glands (Figure 2f,h) were considered to be in a reproductive (i.e., nondiapause) state. The condition of fat body was recorded as either developed (massive or dense; Figure 2a,b,e,f) or loose (poorly developed or depleted; Figure 2c,d,g,h). In total, 696 males and 630 females were dissected (21–45 individuals of each sex from each population per photoperiod).

### 2.4. Statistical Analysis

The duration of the nymphal development was analyzed by multi-way ANOVA followed by Tukey’s HSD test; means and SEM were used as descriptive statistics. Nonparametric data (proportions) were analyzed by multi-way binary probit analysis, the Chi-square test, and the Spearman correlation analysis. Percentage and 95% confidence intervals were used as descriptive statistics. The interrelations between latitude, altitude and photoperiodic threshold were analyzed by GLM.

To estimate the critical day length for the photoperiodic induction of diapause (day length inducing diapause in 50% of individuals), an equation of linear regression was calculated based on the data for 2 photoperiods causing the responses neighboring to 50% (the first—higher than 50%, the second—lower than 50%). Then, based on this equation, the day length corresponding to 50% incidence of diapause was calculated. This method exactly corresponds to the graphical estimation of the critical day length by the point of intersection between the photoperiodic response curve and the level of 50% but provides more accurate results. The correlation between latitude and the critical day length was approximated by linear regression.

All data were analysed with SYSTAT software Version 10.2 (Systat Software Inc., Richmond, CA, USA) [54].

## 3. Results

### 3.1. Nymphal Development

The duration of the *H. halys*’ development from the 2nd nymphal instar to the adult stage significantly depended on the photoperiod (two-way ANOVA, F = 60.2, df = 4, *p* < 0.001) and origin of the *H. halys* population (F = 160.1, df = 3, *p* < 0.001). The interaction between the two factors was also highly significant (F = 14.7, df = 12, *p* < 0.001), indicating that photoperiodic effects on the rate of nymphal development substantially differed among the studied populations. Individuals from all populations showed relatively fast nymphal development under the shortest (L:D 14:10) and the longest (L:D 16:8) photoperiods and a slower development at the intermediate photoperiods.

**Figure 3:**
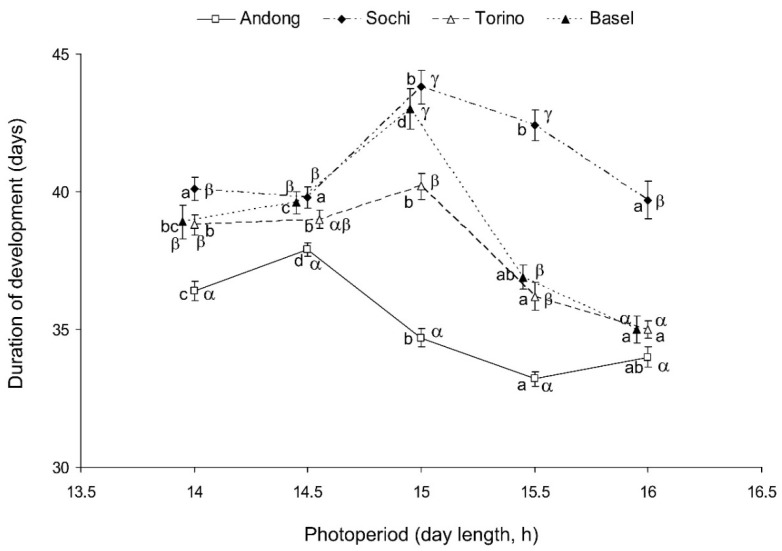
Effect of day length on the duration of nymphal development (2nd instar to adult stage) of *Halyomorpha halys* from four different populations at 24 °C. Means ± SEM are shown (*n* = 61–98 per treatment; data for males and females are combined). Different Latin letters along the same line indicate statistically significant differences between the values for the same population at different photoperiods; different Greek letters within the same day length conditions indicate statistically significant differences between the values for different populations at the same photoperiod (*p* < 0.05; Tukey’s HSD test). Some symbols are slightly shifted horizontally to avoid overlap.

For nymphs of the native (Korean) population, the longest duration of the nymphal period was recorded at L:D 14.5:9.5, whereas for nymphs from the three invasive populations, it was recorded at L:D 15:9 (Figure 3). On average, the development of nymphs from the native population was faster than that of the three invasive populations, and at the photoperiods of L:D 15.5:8.5 and 16:8, the development of the two European invasive populations (Basel and Torino) was faster than that of the Caucasian invasive population (Sochi) (Figure 3).

### 3.2. Development of Reproductive Organs and Fat Body

Degrees of development of reproductive organs and fat bodies strongly negatively correlated both in *H. halys* males (χ^2^ = 224.1, df = 1, *n* = 696, *p* < 0.001, Spearman correlation coefficient *ρ* = –0.567 ± 0.032) and females (χ^2^ = 260.1, df = 1, *n* = 630, *p* < 0.001, *ρ* = –0.643 ± 0.031) (Table 1). Most individuals had either well developed fat bodies and poorly developed reproductive organs or, vice versa, poorly developed fat bodies and well developed reproductive organs, whereas the two other combinations were rarely observed.

The proportion of individuals with a well developed fat body was strongly dependent on the photoperiod, whereas the influence of the population origin was not statistically significant (Table 2). Indeed, although the Chi-square test revealed significant interpopulation differences in fat body development, particularly at the near-threshold photoperiods, no consistent pattern was observed.

**Figure 4:**
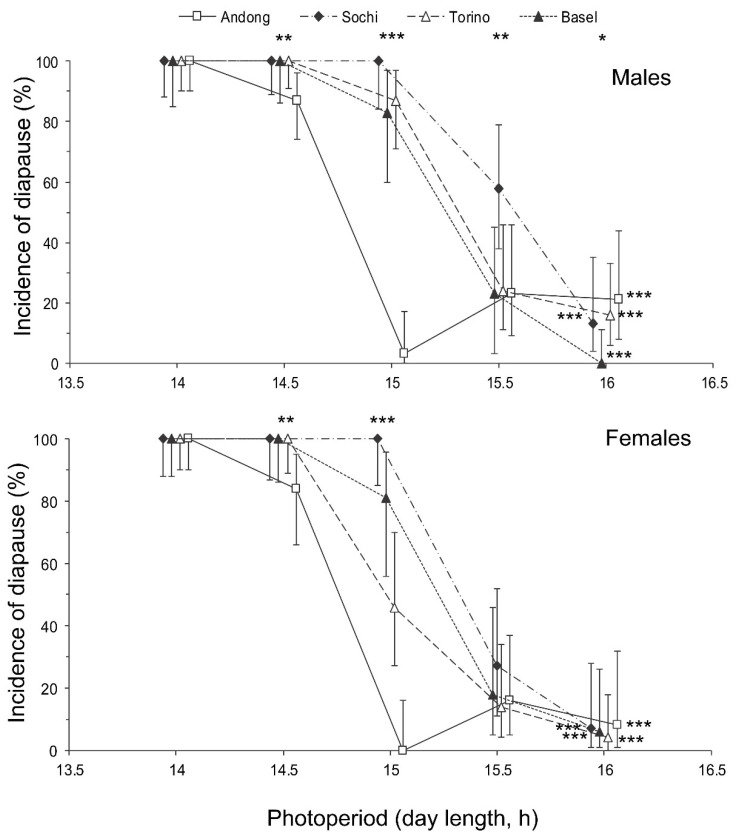
Effect of day length on the incidence of diapause in *Halyomorpha halys* males and females from different populations at 24 °C. Percentage and 95% confidence intervals are shown (*n* = 21–48 per treatment). Asterisks at the right end of the graphs indicate a significant difference between the data for individuals of the same population reared under different photoperiods, i.e., the significant photoperiodic response. Asterisks above the graphs indicate a significant difference between the data for individuals from different populations reared under the same photoperiod, i.e., significant interpopulation variation (* −*p* < 0.05, ** −*p* < 0.01, *** −*p* < 0.001 by the Chi-square test). Some symbols are slightly shifted horizontally to avoid overlap.

**Figure 5:**
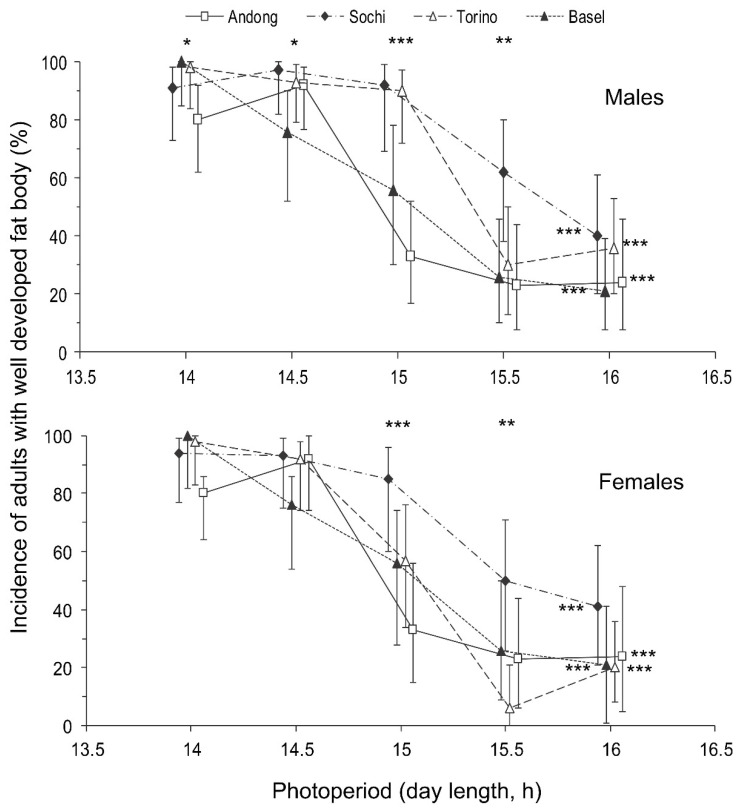
Effect of day length on the development of a fat body in *Halyomorpha halys* males and females from different populations at 24 °C. All other legends are as in Figure 4.

Regarding the photoperiodic responses, the influence of day length on the proportion of individuals with well developed fat bodies was not as clear as that on the proportion of individuals with well developed reproductive organs (Table 2). However, the approximated critical day length of these two photoperiodic responses (i.e., the development of reproductive organs and the development of a fat body) were rather similar among males and females within the same populations: between 14.5 and 15 h in the native (Andong) population and between 15 and 15.5 h in the three invasive populations (Figure 4 and Figure 5).

The proportion of individuals with well developed reproductive organs was strongly dependent on both the photoperiod and population origin with a significant interaction between the two factors (Table 2). Short-day conditions strongly induced diapause in both sexes (Figure 4). The incidence of diapause induction between males and females was not significantly different (Figure 4), as well as the interaction of sex with the photoperiod or population origin (Table 2).

**Figure 6:**
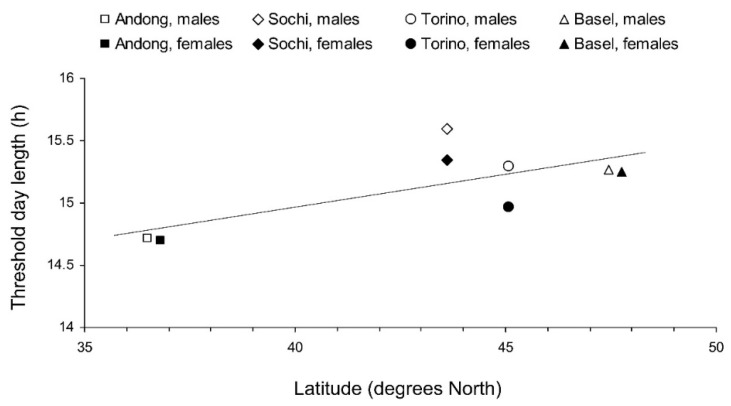
Interrelations between the latitude of the origin and the critical day length for the induction of reproductive diapause in *Halyomorpha halys* adults from different populations at 24 °C. Each symbol represents values for 1 sex from 1 population (Linear regression: Y = 0.052X + 12.9; r = 0.718, *n* = 8, *p* = 0.045). Some symbols are slightly shifted horizontally to avoid overlap.

The interpopulation differences in photoperiodic responses of adult diapause induction were significant (Figure 4). Two-way GLM showed that the critical day length for the induction of diapause (i.e., for the poor development of reproductive organs) in females and males from the four tested populations (*n* = 8) strongly depended on the latitude of the population origin (t = 5.830, *p* = 0.002); the influence of altitude was less strong, although also statistically significant (t = −3.797, *p* = 0.013). However, the dependence on the latitude was determined mostly by the difference between the combined data for invasive (European) and native (Andong) populations, whereas the difference among invasive populations alone did not significantly correlate with latitude (r = 0.375, *n* = 6, *p* = 0.464; Figure 6).

**Figure 7:**
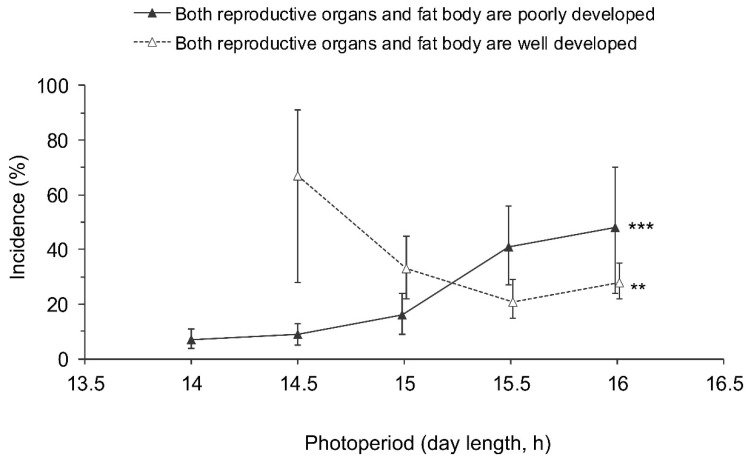
Effect of day length on the incidence of *Halyomorpha halys* adults with poorly developed reproductive organs and fat bodies (among the individuals with poorly developed reproductive organs) and those with well developed reproductive organs and fat bodies (among the individuals with well developed reproductive organs). Percentages and 95% confidence intervals for the pooled data for males and females from all populations are shown (*n* = 12–297 per treatment). Asterisks at the right end of the graphs indicate significant difference between the data for different photoperiods, i.e., significant photoperiodic response (** – *p* < 0.01, *** – *p* < 0.001 by the Chi-square test). Some symbols are slightly shifted along the x-axis to avoid overlap.

Even though adults with well developed reproductive organs and poorly developed fat bodies (Figure 2g,h) and those with poorly developed reproductive organs and a well developed fat body (Figure 2a,b) predominated (Table 1), a few individuals had both fat bodies and gonads well developed (Figure 2e,f) or both systems poorly developed (Figure 2c,d; Table 1). The two latter categories were significantly dependent on the photoperiod, whereas the differences between sexes and populations were not significant (Table 2). Considering the relatively small sample sizes, the data for males and females from all populations were pooled (Figure 7). The proportion of individuals with both poorly developed reproductive organs and fat bodies increased with the photoperiod, whereas the proportion of individuals with both well developed reproductive organs and fat bodies tended to decrease with increasing day length (Figure 7).

## 4. Discussion

The photoperiodic response of the diapause induction of individuals from the native Korean population (the critical day length between 14.5 and 15.0 h) was similar to that of the populations from Japan (the critical day length between 13.5 and 15.0 h [27,28]). The photoperiodic response of the Sochi population (the critical day length between 15.0 and 15.5 h) corresponded well to the results of the previous study of the same population at the same temperature (between 15.0 and 16.0 h) [26] considering that in the present work we used a two-times finer scale.

A pronounced correlation between the critical photoperiod of the winter diapause induction and geographic latitude of origin is a fundamental characteristic of multivoltine insect species with wide ranges [8,11,12,13,15]. It has been demonstrated in several heteropterans [47,48,55,56,57,58], although in most of these studies only two or three populations were compared, whereas our experiments were conducted on representatives of four populations.

A meta-analysis of studies on numerous insects from different orders suggested that with a 5° change in latitude, the critical photoperiod for diapause induction changes by an average of 1.5 h [12,13,59]. The difference between geographic latitudes of invasive *H. halys* populations from Sochi and Basel that were used in our study was close to 5°, however, the expected corresponding difference between critical photoperiods was not observed. Moreover, the difference between critical photoperiods of native Korean and invasive European populations was much smaller than the expected 3 h difference based on the 10° difference in latitude. The discrepancy between our data and the above-mentioned generalization of the earlier studies is likely influenced by a very rapid spread of the brown marmorated stink bug on the European continent. Consequently, natural selection may not have happened yet to ensure ‘instant adaptation’ of the invader to new environments. Somewhat similar results were reported for the invasive ladybird, *Harmonia axyridis* (Pallas) (Coleoptera: Coccinellidae). Instead of a rapid adaptation of the photoperiodic response to the critical day length of the newly invaded region, the invasive populations of the beetle decreased their dependence on day length and shifted to a diet-induced diapause [18]. On the other hand, a period of 20 years was sufficient for the rapid adaptive evolution of the photoperiodic response of the invasive Asian tiger mosquito *Aedes albopictus* Scuse (Diptera: Culicidae) [17]. Evidently, the rate of microevolution of the ecophysiological control of seasonal cycles depends on various factors, such as initial intrapopulation variability, degree of environmental novelty, and selection pressure.

On average, the duration of *H. halys* nymphal development observed in our experiment was similar to the results of earlier studies [28,29,31,32,35,60]. The photoperiodic impact on the growth rate and development of true bug species has been demonstrated in many cases [29,53,61,62,63,64,65,66,67,68,69]. In insects with a long-day type photoperiodic response, short-day conditions often accelerate the development of pre-diapause stages and thus increase the chances of individuals to timely enter diapause. However, in *H. halys*, another pattern of the response was observed: the rates of nymphal development were relatively high at both short and long photoperiods and significantly decreased at the intermediate (near-critical) day lengths. Similar results were obtained for the linden bug, *Pyrrhocoris apterus* (L.) (Hemiptera: Heteroptera: Pyrrhocoridae) [61,62] and the ground cricket, *Dianemobius nigrofasciatus* Walker (Orthoptera: Gryllidae) [70], and the authors suggested that deceleration of development at the near-critical photoperiods gives individuals more time for a ‘fine-tuning’ of the diapause-inducing response.

Remarkably, in *H. halys*, the interpopulation differences in the patterns of photoperiodic effects on the rates of nymphal development correlated with the corresponding differences in diapause-inducing photoperiodic responses. In all populations tested, deceleration of development was observed at the corresponding near-critical photoperiods, suggesting that this correlation was not an occasional coincidence but an important (likely adaptive) species-specific feature, which was not lost during the invasion of the European continent. Photoperiodic effects on the rate of larval development and on the induction of diapause also correlated in the pitcher-plant mosquito, *Wyeomyia smithii* (Coquillett) (Diptera: Culicidae) [71]. Similar results were obtained in experiments with some other insects [72,73,74]. However, a comparative study of four populations of another rapidly spreading invader, *H. axyridis,* showed that microevolutionary changes in the two photoperiodic responses influencing larval development and female maturation did not correlate [19].

## 5. Conclusions

In summary, our hypothesis that differences between photoperiodic responses of distant *H. halys* populations would correlate to a geographic latitude was only partially confirmed. The interpopulation differences in critical photoperiods for diapause induction and the duration of nymphal development of *H. halys* significantly correlated with latitude, however, these correlations were rather weak and mostly determined by the difference between native Korean and invasive European populations. Differences between the three European populations were not significant, although they originated from far apart regions. The latter likely indicates a very rapid continent-wide invasion of the brown marmorated stink bug: the microevolution was likely ‘too slow to keep up’ with the rapid spread of the invader across wide regions with different climates. Moreover, the difference between Korean and European populations was much smaller than expected based on the analysis of the data available for other widely distributed insects. Hence, it can be expected that in the near future, the critical photoperiods of invasive *H. halys* populations will gradually change in accordance with the peculiarities of local climates. At present, however, the same critical day length for diapause induction (about 15 h 15 min) can be used for all European *H. halys* populations to model phenology, further spread, and their response to climate change.

## Figures and Tables

**Table 1:** The correlation between the development of a fat body and reproductive organs in *Halyomorpha halys* males and females (percentages calculated for the pooled data of all photoperiods and populations: 696 males and 630 females; see text for details of statistical analysis).

Fat Body	Reproductive Organs			
	Poorly Developed		Well Developed	
	Males	Females	Males	Females
Poorly developed	9.0	6.3	26.6	31.1
Well developed	53.3	51.6	11.1	11.0

**Table 2:** Photoperiodic effects on the development of reproductive organs and fat bodies in *Halyomorpha halys* males and females from four different populations (Binary probit analysis (0—no, 1—yes): regression coefficient C ± SE and significance (*p*) of influence).

Factor or Combination of Factors and Coding	Influence on the Proportion of Individuals with Well Developed Reproductive Organs ^1^	Influence on the Proportion of Individuals with Well Developed Fat Body ^1^	Influence on the Proportion of Individuals with Both Well Developed Reproductive Organs and Fat Body ^2^	Influence on the Proportion of Individuals with Both Poorly Developed Reproductive Organs and Fat Body ^3^
Photoperiod (day length)	1.0 ± 0.2, *p* < 0.001	–1.3 ± 0.2, *p* < 0.001	–0.9 ± 0.3, *p* = 0.010	0.9 ± 0.3, *p* < 0.001
Population (1—Andong, 2—Torino, 3—Basel, 4—Sochi)	–9.3 ± 1.7, *p* < 0.001	–0.5 ± 0.8, *p* = 0.588	–3.7 ± 2.4, *p* = 0.128	0.6 ± 1.3, *p* = 0.630
Sex (0—males, 1—females)	–4.2 ± 3.2, *p* = 0.192	0.8 ± 1.8, *p* = 0.676	–4.3 ± 4.7, *p* = 0.369	–0.3 ± 3.1, *p* = 0.928
Population × photoperiod	0.6 ± 0.1, *p* < 0.001	0.0 ± 0.1, *p* = 0.425	0.2 ± 0.2, *p* = 0.118	–0.1 ± 0.1, *p* = 0.561
Population × sex	0.0 ± 0.1, *p* = 0.958	0.0 ± 0.1, *p* = 0.955	0.1 ± 0.1, *p* = 0.658	0.1 ± 0.1, *p* = 0.254
Sex × photoperiod	0.3 ± 0.2, *p* = 0.171	–0.1 ± 0.1, *p* = 0.649	0.3 ± 0.3, *p* = 0.404	0.0 ± 0.2, *p* = 0.971

^1^ Among all individuals (*n* = 1326). ^2^ Among individuals with well developed reproductive organs (*n* = 527). ^3^ Among individuals with poorly developed reproductive organs (*n* = 799).

## Data Availability

Data supporting reported results can be obtained upon request from the corresponding author (D.L.M.).
